# Processed Food–Sweets Patterns and Related Behaviors with Attention Deficit Hyperactivity Disorder among Children: A Case–Control Study

**DOI:** 10.3390/nu15051254

**Published:** 2023-03-02

**Authors:** Wu Yan, Shuang Lin, Dandan Wu, Yanan Shi, Lihua Dou, Xiaonan Li

**Affiliations:** 1 Department of Children Health Care, Children’s Hospital of Nanjing Medical University, Nanjing 210008, China; 2 Institute of Pediatric Research, Nanjing Medical University, Nanjing 210029, China

**Keywords:** ADHD, dietary patterns, eating behavior, children, factor analysis

## Abstract

Background: Previous studies have focused on the associations between core symptoms and dietary intake in children with attention deficit hyperactivity disorder (ADHD). However, few studies have explored how dietary patterns and behaviors relate to the risk of ADHD. The aim of our study is to explore the associations between dietary patterns and behaviors and the risk of ADHD, which could provide evidence for follow-up and treatments for children with ADHD. Methods: We conducted a case–control study, including 102 children diagnosed with ADHD and 102 healthy children. The food frequency questionnaire (FFQ) and the children’s eating behavior questionnaire (CEBQ) were used to investigate food consumption and eating behaviors. We applied exploratory factor analysis for constructing dietary patterns, and the factor scores were adopted for log-binomial regression to assess the associations between how dietary patterns and eating behaviors affect the risk of ADHD. Results: We extracted five dietary patterns with a cumulative contribution rate of 54.63%. Processed food–sweets scores revealed positive associations with an increased risk of ADHD (OR = 1.451, 95% CI: 1.041–2.085). Moreover, processed food–sweets tertile 3 was associated with an increased risk of ADHD (OR = 2.646, 95% CI: 1.213–5.933). In terms of eating behaviors, the group with a higher score relating to a desire to drink was also positively correlated with the risk of ADHD (OR = 2.075, 95% CI: 1.137–3.830). Conclusions: In the treatment and follow-up of children with ADHD, dietary intake and eating behaviors should be considered.

## 1. Introduction

Attention deficit hyperactivity disorder (ADHD) is one of the most common neurodevelopmental disorders in childhood, and it is characterized by age-inappropriate inattention, hyperactivity, and impulsivity [1]. It has been reported that the prevalence of ADHD in children around the world is about 5.9–7.1%, and the trend is increasing year by year [2]. In China, almost 23 million children and adolescents suffer from ADHD, with a prevalence rate of 6.26% [3]. Children with ADHD may affect their family relationships, school performance, and social interactions [4]. In addition, the core symptoms may persist into adolescence or even adulthood. Studies have shown that 30–70% of patients may still have significant symptoms into adulthood [5,6], seriously impacting and heavily burdening patients, families, and society. Given that, ADHD has attracted extensive attention in the field of medicine and public health worldwide.

It is worth noting that children with ADHD are more likely to suffer from dietary intake problems, such as unreasonable nutrient intake, disordered dietary structures, and poor eating behaviors [7,8]. Dietary patterns and nutrient intake may be associated with the increased or decreased risk of ADHD in children. A meta-analysis based on 14 studies suggested that a diet high in refined sugar and saturated fat increases the risk of disease, whereas diets characterized by high consumption of fruits and vegetables reduce the risk of ADHD [8]. Among them, as a combination of diets and nutrients with different characteristics, dietary patterns represent a wider range of nutrient consumption. A study on Chinese children found that fish–white meat diets and algae–mineral protein nutrition patterns may effectively control ADHD in children [9]. A case–control study suggested that a Western diet or low adherence to a Mediterranean diet was positively associated with ADHD symptoms [10]. A study conducted in South Korea showed that the high consumption of traditional healthy dietary patterns and the low consumption of snack patterns of fast food and beverages were negatively correlated with ADHD in school-age children [11]. Therefore, providing suitable dietary patterns is beneficial in the treatment and follow-up of ADHD.

As a significant approach for children to receive nutrition, food intake can be considered largely affected by eating behaviors. Available evidence shows that children with ADHD are accompanied by more unhealthy eating behaviors, and emotional or overeating may be triggered by hyperactivity or impulsivity. For example, food addiction/overeating was significantly associated with ADHD diagnoses in both children and adults and may be an important variable in the relationship between ADHD and obesity [12]. Addiction-like eating behaviors are associated with an excessive intake of high-fat foods and/or refined carbohydrates, further contributing to overweight and obesity in children [13]. In addition, children with ADHD and poor eating behaviors also have increased rates of comorbid disorders in adulthood [14]. A cross-sectional study conducted in the United States showed that inattention and hyperactivity/impulsivity in preschool children were positively associated with food responsiveness, emotional overeating, and slow eating [15]. These studies emphasize the relationship between children’s ADHD symptoms and eating behavior, even physical development, and also illustrate the importance of healthy eating behaviors. It is well known that eating behaviors during childhood are the basis of dietary consumption and nutrition acquisition. Poor eating behaviors may lead to unbalanced dietary nutrient intake and even affect growth and development. However, dietary intake and dietary-behavior-related issues are usually ignored in the treatment and follow-up of children with ADHD.

In this research, we designed a case–control study and applied factor analysis to investigate the associations between dietary patterns and eating behaviors with the risk of ADHD. We hypothesized that certain dietary patterns or behaviors might be associated with the increased risk of ADHD. Our study not only provides evidence for the follow-up and treatments of ADHD, but also supports references for correcting growth deviations in children with ADHD.

## 2. Materials and Methods

### 2.1. Study Population

This is a case–control study with a matching design that includes children who visited the Department of Children Health Care from June 2020 to December 2020. The case group included 102 children who were diagnosed with ADHD by the attending physicians or above in the psychological–behavioral clinic. The inclusion criteria for the case group are as follows: (1) age 6–13 years. (2) First visit and meeting the diagnostic criteria for the fifth edition of the Diagnostic and Statistical Manual of Mental Disorders (DSM-V) [1]. (3) No history of anti-ADHD medication. The exclusion criteria are as follows: ADHD symptoms caused by other neurodevelopmental disorders, psychotic disorders, mood disorders, medications, or organic diseases. The control group was matched with healthy children who underwent physical examination during the same period. They also completed the DSM-V assessment and ruled out ADHD. The protocol was approved by the Medical Ethics Committee of the Children’s Hospital of Nanjing Medical University (Ethics number: NMUB2018074). The children and their parents participated in the assessment voluntarily, and written informed consent was obtained from their parents.

### 2.2. Dietary and Behavioral Assessment

In this study, food frequency questionnaires (FFQs) were used to investigate the frequency and total intake of various food types. The questionnaires were designed by a panel of experts, including scientists in the fields of epidemiology and nutrition [9]. In the published children’s studies, the FFQ has been recognized as a reliable tool for collecting data with good validity and reliability [16,17]. It is widely used in epidemiological studies concerning the association between dietary intake and health outcomes. A semi-quantitative food frequency questionnaire was used to obtain children’s food consumption in the last month [18], including 18 food categories such as rice, coarse grains, vegetables and fruits, meat, fish and shrimp, snacks, sugary drinks, and so on. According to the frequency of food consumption, food consumption was classified as follows: 2 or more times a day, once a day, 4–6 times a week, 2–3 times a week, once a week, 2–3 times a month, once a month, less than once a month. Photographs of food portion sizes were presented to assist in estimating food consumption. The product of the participants’ single intake and daily consumption frequency was the total daily intake. The total daily intake was presented in grams (g) or milliliters (mL) by household measurement. Furthermore, the daily dietary energy intake and nutrient intake of children were analyzed using a 24 h dietary review method and dietary nutrition analysis software, Nutrition Star (Child care V 5.3.0) (ShangHai ZhenDing Computer Science Technology Co., Ltd., Shanghai, China).

In addition, the Children’s Eating Behavior Questionnaire (CEBQ), developed by Jane Wardle et al., was used to evaluate children’s eating behaviors [19]. It is widely used to evaluate eating behaviors aged 2–13 years old, and has achieved good internal consistency, reliability, and validity in various studies [20,21]. There are 35 questions in the questionnaire, which are divided into 8 types of eating behaviors, including the food avoidance dimension (satiety responsiveness, slowness in eating, food fussiness, emotional undereating) and food tendency dimension (food responsiveness, enjoyment of food, desire to drink, emotional overeating). The questionnaire is based on a 5-point Likert scoring method (never = 1; rarely = 2; sometimes = 3; often = 4; always = 5). In general, a higher score indicates more serious eating behavior problems.

### 2.3. Covariates

Physical measurement technicians measured the height and weight of the children and the parents, with an accuracy of 0.1 cm for height and 0.01 kg for weight, and the body mass index (BMI) was calculated. In addition, the participants completed the self-designed questionnaires under the guidance of the investigators, which included demographic characteristics, parents’ education level, annual family income, and the parent–child relationship. These covariates were taken into account in the analysis.

### 2.4. Statistical Analysis

Epidata3.1 was used to input the questionnaire and scale the data. Continuous data that followed a normal distribution are expressed as mean ± standard deviation, and an independent sample t-test was used to compare the differences between the subjects with ADHD and the controls. The non-normal continuous variables are expressed as median (P_25_, P_75_), and the Mann–Whitney U test was used to compare the difference between the groups. The qualitative data are represented by frequency (%), and s Chi-square test was used to compare the groups.

Exploratory factor analysis was used to construct the dietary patterns of 18 food consumption categories obtained by the FFQ. It is mainly based on the principle of dimensionality reduction, exploring the correlation coefficient matrix between different food types and grouping by the correlation size of various foods so that the correlation between the foods in the same dietary pattern is high, while the correlation between the foods in different dietary patterns is low. Firstly, the Kaiser–Meyer–Olkin (KMO) test and Bartlett sphericity test were performed to evaluate whether factor analysis was appropriate (KMO > 0.6, *p* < 0.05). Secondly, common factors were extracted based on principal component analysis, and five common factors were determined based on the dietary pattern, with a factor load coefficient > 0.35. The extracted common factors were further rotated by the maximum variance method to achieve a simpler structure and better interpretation, where a positive loading indicates a positive association between a given food and the pattern, while a negative loading indicates a negative association with the pattern. The factor loading represents the correlation coefficient between each food and the dietary pattern, reflecting the importance of each food in the dietary pattern; that is, foods with a high absolute load are considered to be the main factor of the dietary pattern. Next, the variance contribution rate of each factor was calculated, which is an index to measure the importance of the common factors.

In addition, factor scores were calculated to evaluate the participant’s score on each common factor, which was the outcome of the dimensionality reduction of the original variable and could be used for regression analysis. Log-binomial regression was used to examine the association between the different dietary pattern factor scores (continuous, after calculating the tertile) and the odds of ADHD. The tertiles divide a sequence of numbers into three equal parts, including low-value (Tertile 1), medium-value (Tertile 2), and high-value (Tertile 3) groups. A *p*-value of <0.05 based on 2-tailed test results was considered statistically significant. All of the analyses were performed with R version 3.2.2.

## 3. Results

### 3.1. Characteristics of Participants

Table 1 shows the characteristics of the children included in the study. There were 102 children in the ADHD group and an equal number in the control group, which included 67 males and 35 females. There were no significant differences in age, gender, BMI, and daily screen time between the two groups. In addition, there were also no significant differences in family characteristics such as parents’ BMI, parents’ education, and annual family income, but there were significant differences found in the parent–child relationship between the two groups (Table 1).

### 3.2. Dietary Patterns Extraction

The KMO statistic was 0.707, and the Bartlett sphericity test was *p* < 0.05, indicating that there was a significant correlation between the analyzed variables; therefore, it is suitable for factor analysis. A total of five dietary patterns were extracted, namely coarse grains–poultry–vegetables (tubers, bean products, coarse grains, poultry and meat, and vegetables), processed food–sweets (processed meat, fried food, puffed food, sugared beverages, and candies), dairy–seafood (milk and dairy products, fish, and prawns), fruits–nuts (mushrooms and seaweed, nut, and fruits), and staple foods (eggs, rice products, and flour products). The variance contribution rate of each factor was calculated by dividing the square sum of each factor load by the number of variables, which was used to measure the importance of common factors. Overall, the five dietary patterns cumulative variance accounted for 54.63% (Table 2).

### 3.3. Associations between Dietary Patterns and ADHD

**Figure 1:**
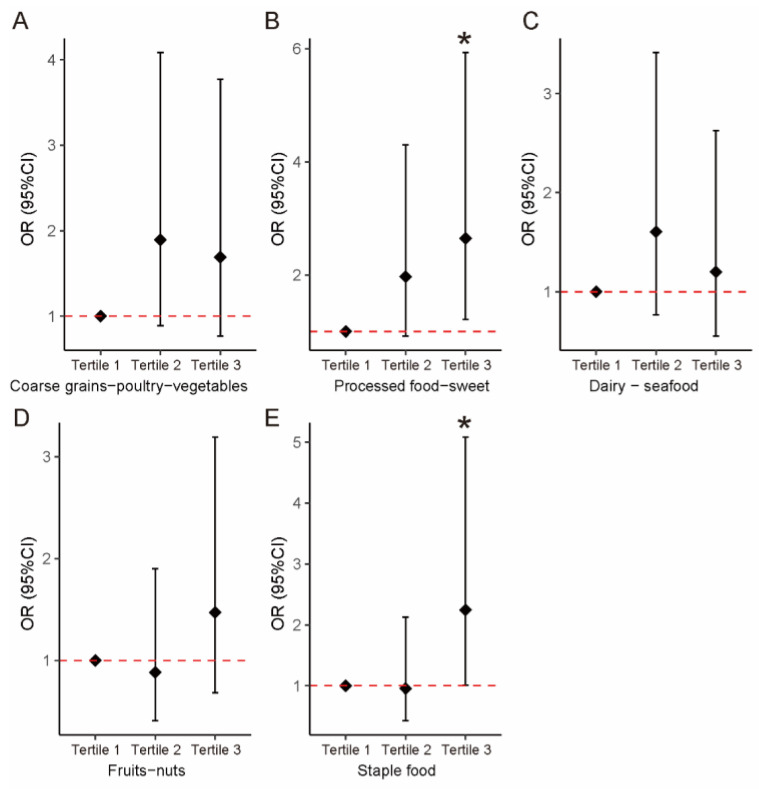
Association between the tertiles dietary patterns and the risk of ADHD. (**A**) Coarse grains–poultry–vegetables. (**B**) Processed food–sweets. (**C**) Dairy–seafood. (**D**) Fruits–nuts. (**E**) Staple food. Solid diamonds and horizontal lines indicate OR and 95% CI, respectively. * *p* < 0.05.

Log-binomial regression was used to investigate the association between dietary patterns and the risk of ADHD diagnosis. Model 1 was an unadjusted model, and model 2 was further adjusted for covariates. The results showed that there were significant positive associations between the processed food–sweets scores and the diagnosis of ADHD (OR = 1.451, 95% CI: 1.041–2.085). In addition, when the scores were converted to tertiles, the risk of ADHD was increased in the tertile 3 group (OR = 2.646, 95% CI: 1.213–5.933). We also found that the risk of ADHD was significantly increased in the tertile 3 group with staple food patterns (OR = 2.246, 95% CI: 1.013–5.085) (Table 3) (Figure 1).

### 3.4. Comparison of Eating Behaviors

Since the eating behavior scores did not conform to a normal distribution, the Mann–Whitney U test was applied to analyze the differences between the ADHD and control groups. No differences were found between the two groups of children in terms of food-avoidant behaviors, while regarding the food approach dimension, ADHD children showed significantly higher “desire to drink” scores compared to the controls (Table 4).

### 3.5. Associations between Eating Behaviors and ADHD

**Figure 2:**
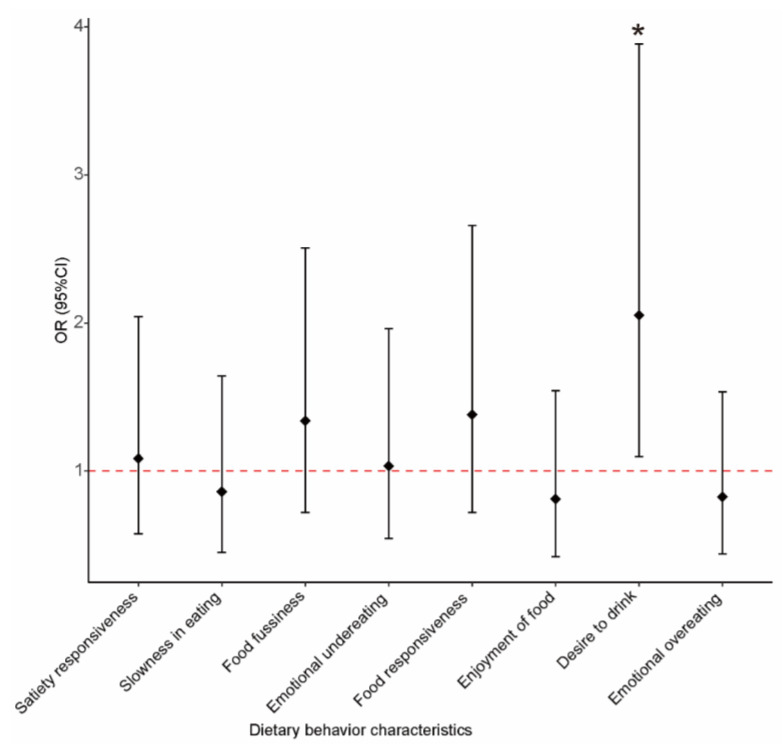
Association between the median-based groups of eating behavior characteristics and the risk of ADHD. Solid diamonds and horizontal lines indicate OR and 95% CI, respectively. * *p* < 0.05.

In addition, the association between eating behavior characteristics and ADHD risk was examined, and no significant association was found between the eating behavior scores and ADHD risk. The children were divided into two groups based on the median score; the risk of ADHD was associated with the upper median group on the dimension of “desire to drink”, and there were no differences in the other dimensions (Table 5) (Figure 2).

### 3.6. Comparison of Daily Nutrient Intake

The daily intakes of nutrients obtained from the 24 h dietary review were compared between the two groups. It was found that the daily intakes of energy, fat, carbohydrate, iodine, and nicotinic acid in the ADHD group were significantly higher than those in the control group (Table S1).

## 4. Discussion

In our study, five mainstream dietary patterns were identified to investigate eating behaviors in children. A processed food–sweets pattern rich in processed meat, fried food, puffed food, sugared beverages, and candies was positively associated with ADHD. The staple food pattern of flour products, rice products, and eggs was also associated with an increased risk of ADHD. In addition, in terms of eating behavior, the desire to drink in the food approach dimension was also positively associated with ADHD. Therefore, the findings validated the consistency of processed food–sweets dietary pattern and desire to drink behavior. To our knowledge, we are the first group to combine dietary patterns and eating behaviors to explore their relationship with ADHD after identifying risk factors separately and then mutually validating each factor. Clinicians and researchers are expected to explore comprehensive interventions for dietary structure and behaviors in order to achieve greater benefits.

Our study found that processed food–sweets scores were associated with the risk of ADHD diagnosis. Similarly, in a study of Iranian children, adherence to fast food and sweet dietary patterns was associated with a higher prevalence of ADHD [22], and similar results were found in Spain [10]. Sugar consumption is considered to be closely related to ADHD. After sugar enters the blood, it causes rapid changes in glucose levels and produces more adrenaline, which not only provides more short-term energy for physical activities but also shows more excitement or impulsiveness. Even healthy children who ingest high doses of sugar on an empty stomach produce high levels of adrenaline, which in turn can cause tremors, anxiety, excitement, and poor concentration [23]. In addition, sugar-rich foods could trigger the reward system [24,25], and neuroimaging studies suggest that the neurobiological mechanisms of ADHD involve reward system dysfunction [26,27]. The reward system is known as the pleasure center of the brain, the most important of which is the dopamine system [28]. Normally, the dopamine released by the nerve impulse is quickly reabsorbed in equal amounts, making the body produce pleasure and the brain receives a “reward”. The intake of sugar would activate the central reward system, induce the release of dopamine and produce a rewarding effect, as well as produce memories related to the sugar reward. In addition, it induces the body to ingest sugar again, even producing addictive behaviors, and long-term addictive behaviors overstimulating the brain and leading to self-protection, reducing the sensitivity of the dopamine receptors [29]. We also found that excessive staple food intake was associated with the risk of ADHD. Children with ADHD are often troubled with characteristics such as impulsivity and emotional instability, which may lead to poor eating behaviors, such as being picky, a desire to drink, etc., and ultimately more high-fat and/or refined carbohydrate foods [13]. These foods balance mood disorders as a form of self-treatment to modulate disturbances of dopamine metabolism and reward–punishment effects [30].

The daily intakes of energy, carbohydrate, and fat in the ADHD group were significantly higher than those in the control group, which was consistent with the “processed food-sweet” dietary pattern. Carbohydrates include all kinds of sugars, and the intake of sweets may account for a large proportion in this population. In addition, processed food is often high in energy and fat, which may be associated with increased daily energy and fat intake in children with ADHD [31]. In addition, iodine is the main raw material for synthesizing thyroid hormones, which is closely related to the neurobehavioral development of children [32]. A Norwegian cohort study found that insufficient iodine intake by mothers during pregnancy was associated with increased ADHD symptom scores in children [33]. In our study, the increased daily iodine intake of ADHD children may be related to seafood consumption. Finally, nicotinic acid is an essential nutrient for humans and animals. Liver, kidney, yeast powder, and wheat germ are rich in nicotinic acid [34]. Nicotinic acid has been shown to be effective in alleviating anxiety and depression [35], but its effect remains unclear in children with ADHD. A review that critically appraised the effects of different dietary therapies on ADHD suggested that restricted elimination diets may be beneficial, artificial food coloring elimination was a potentially valuable treatment method, and that additional dietary supplements may also achieve some positive results, but a larger sample size and an evaluation of long-term results are still needed to determine the potential value [36]. A narrative review suggested that vitamin D and magnesium supplementation appeared to improve behavioral function and mental health in children with ADHD [37]. Additionally, blood zinc levels were negatively associated with ADHD [9], and zinc supplementation could reduce ADHD symptoms in children with zinc deficiency [38]. In contrast, biologics, particularly Lactobacillus rhamnosus GG, do not have sufficient evidence to be recommended as probiotic supplements for the treatment of ADHD [37]. The study provides new ideas for the relationships between ADHD and nutrient intake, and more research is needed to verify these findings in the future.

The FFQ was used to obtain the frequency and quantity of various foods consumed by ADHD children before the study period. It has the advantage of understanding dietary patterns and habits compared with short-term diet reviews. However, the results may not be accurate due to the fact that the report is recalled. The 24 h dietary review was a dietary log filled by the ADHD children and parents. The weight of various foods was calculated by the nutritionist, and the dietary nutrition analysis software calculated the energy intake and nutrient content of children’s daily diets, which may be more accurate. Therefore, the two evaluation methods were combined in our study. Among the evidence between eating behaviors and ADHD diagnosis, a longitudinal study suggested that food responsiveness was an early marker of ADHD symptoms at 6 years of age [39]. In contrast, children with ADHD also tend toward more dietary, behavioral problems [8], similar to the “desire to drink” problem we found; therefore, physical growth deserves attention. In our study, the BMI of ADHD children showed an increasing trend, even if there was no statistical difference. A meta-analysis based on 42 studies found that the proportion of obesity in children with ADHD increased by 40% compared with the control group [40]. A cohort study also found that children with ADHD at the age of 6 years had a significant increase in body fat content after 3 years of follow-up compared with the control children [41]. In addition, some studies have shown that the proportion of height and weight loss in ADHD children is significantly higher than that of the control group [42], which means that ADHD children face more problems in terms of growth deviation. Therefore, the clinical treatment and follow-up of ADHD should not only focus on improving the patient’s symptoms but also regularly evaluate dietary intake and behavior and monitor physical growth.

The major strengths of this study included the fact that the ADHD cases were first-time visits without any medication or other intervention, as the use of certain medications would affect children’s appetite, which would also lead to changes in dietary intake. Secondly, all of the assessments were conducted on outpatients by trained psychologists and dietitians, with high compliance and producing good-quality information. Thirdly, the FFQ combined with factor analysis summarized dietary patterns, which represented a broader picture of food and nutrient consumption, so it was more valuable than single food or nutrition in predicting disease risk, which also showed that the results were reliable and stable. Finally, it was found that the “processed food-sweet” dietary patterns and “desire to drink” eating behaviors were both associated with the risk of ADHD in children.

There were some limitations in our study. Due to the cross-sectional design, the causal relationships between dietary intake, behavior, and a diagnosis of ADHD could not be obtained, and patients with eating disorders could not yet be identified, as these need to be confirmed in a longitudinal study. Other possible confounders associated with ADHD and dietary intake, such as labor complications, breastfeeding, and household composition, cannot be ignored [9] and require further confirmation in subsequent studies. In addition, the population included in the study were school-age children, which some of meals were provided at school. Parents may be less aware of the number of school meals their child receives, and their reported dietary intake may be biased, so self-reported information from the children was also collected. A retrospective recollection of dietary information over the past month may be challenging, and it is essential to incorporate daily dietary reviews. The sample size of this study may limit the power of our evidence to some extent. Finally, the factor analysis cumulative variance was 54.63%, indicating other dietary patterns that have not been extracted; however, these values should be interpreted with caution as it depends on the number of variables included in the factor analysis.

## 5. Conclusions

This case–control study found that processed food–sweets and staple foods were significantly associated with an increased the risk of ADHD, as was the desire to drink behavior. Therefore, improving health education related to eating behaviors and dietary patterns might be an effective and practical method for ADHD prevention and control among Chinese children. It is worth further investigating causality and determining whether dietary manipulation helps improve the core symptoms. In summary, we suggest that attention should be paid to diet and behavioral management in the treatment and follow-up of children with ADHD to reduce the risk of dietary factors on the core symptoms of ADHD as well as growth and development.

## Figures and Tables

**Table 1:** Characteristics of participants in ADHD and control groups.

Characteristics	ADHD (n = 102)	Controls (n = 102)	*t*/*χ*^2^	*p*-Values ^1^
Age (year)	8.90 ± 1.57	8.94 ± 1.93	0.179	0.858
Gender				
Male	67 (65.69)	67 (65.69)	0	1.000
Female	35 (34.31)	35 (34.31)		
BMI (kg/m^2^)	17.36 ± 3.62	16.66 ± 3.25	−1.444	0.150
Daily screen time (h)	1.06 ± 0.90	1.09 ± 1.02	0.243	0.808
Paternal BMI (kg/m^2^)	24.48 ± 3.07	25.03 ± 3.31	1.236	0.218
Maternal BMI (kg/m^2^)	22.96 ± 3.72	22.21 ± 3.07	−1.756	0.081
Paternal education levels				
Junior high and below	63 (61.76)	65 (63.73)	0.428	0.934
Technical secondary or senior high school	15 (14.71)	14 (13.73)		
College or Bachelor degree	17 (16.67)	18 (17.65)		
Master degree or above	7 (6.86)	5 (4.90)		
Maternal education levels				
Junior high and below	73 (71.57)	67 (65.69)	7.169	0.067
Technical secondary or senior high school	13 (12.75)	6 (5.88)		
College or Bachelor degree	13 (12.75)	26 (25.49)		
Master degree or above	3 (2.94)	3 (2.94)		
Family income				
<150,000 yuan	51 (50.00)	61 (59.80)	1.980	0.159
≥150,000 yuan	51 (50.00)	41 (40.20)		
Parent–child relationship				
Authoritarian	62 (60.78)	54 (52.94)	11.186	**0.011**
Doting	9 (8.82)	6 (5.88)		
Laissez-faire	16 (15.69)	8 (7.84)		
Democratic	15 (14.71)	34 (33.33)		

ADHD, attention deficit hyperactivity disorder; BMI, body mass index. *p*-Values < 0.05 are bolded. ^1^ *p*-values from *t* tests or *χ*^2^ tests.

**Table 2:** Rotated component matrix for the five identified dietary patterns in the study children.

Food Groups	Dietary Patterns ^1^				
	Factor 1	Factor 2	Factor 3	Factor 4	Factor 5
	Coarse Grains–Poultry–Vegetables	Junk Food–Sweets	Dairy–Seafood	Fruits–Nuts	Staple Food
Tubers	**0.892**	0.002	0.046	0.031	0.018
Bean products	**0.883**	0.005	−0.019	0.125	−0.044
Coarse grains	**0.872**	−0.029	−0.004	0.026	−0.096
Poultry and meat	**0.639**	0.166	0.418	−0.045	0.164
Vegetables	**0.590**	−0.063	0.112	0.319	0.325
Processed meat	0.099	**0.719**	−0.083	−0.067	−0.230
Fried food	0.065	**0.682**	0.256	−0.052	0.073
Puffed food	0.050	**0.667**	−0.129	0.054	−0.043
Sugared beverages	−0.108	**0.499**	−0.04	0.117	0.235
Candies	−0.129	**0.490**	−0.006	0.324	−0.100
Milk and dairy products	0.096	−0.147	**0.799**	0.042	−0.183
Fish and prawn	0.041	0.038	**0.735**	0.042	0.163
Mushrooms and seaweed	0.145	0.081	−0.079	**0.679**	0.171
Nut	−0.061	0.149	0.085	**0.585**	−0.060
Fruits	0.333	−0.172	0.060	**0.573**	−0.160
Eggs	0.072	−0.177	0.027	0.165	**0.671**
Rice products	0.065	0.172	0.062	−0.122	**0.597**
Flour products	0.212	0.185	0.185	0.277	**−0.395**

^1^ The factor-loading scores with absolute values of ≥0.35 are bolded.

**Table 3:** Associations between dietary patterns factor scores and tertiles and the risk of ADHD.

Dietary Patterns	Model 1 ^1^			Model 2 ^2^		
	OR	95% CI	*p*-Values	OR	95% CI	*p*-Values
	Factor 1: Coarse grains–poultry–vegetables					
Continuous	0.949	(0.653, 1.294)	0.733	0.992	(0.648, 1.357)	0.959
Tertile 1	Ref			Ref		
Tertile 2	2.192	(1.076, 4.542)	**0.032**	1.893	(0.888, 4.084)	0.100
Tertile 3	1.802	(0.886, 3.711)	0.106	1.691	(0.767, 3.770)	0.194
	Factor 2: Processed food–sweets					
Continuous	1.365	(1.010, 1.895)	**0.049**	1.451	(1.041, 2.085)	**0.035**
Tertile 1	Ref			Ref		
Tertile 2	1.689	(0.83, 3.474)	0.150	1.969	(0.919, 4.301)	0.084
Tertile 3	2.343	(1.149, 4.869)	**0.021**	2.646	(1.213, 5.933)	**0.016**
	Factor 3: Dairy–seafood					
Continuous	0.990	(0.740, 1.324)	0.946	1.046	(0.757, 1.445)	0.785
Tertile 1	Ref			Ref		
Tertile 2	1.475	(0.728, 3.011)	0.282	1.605	(0.765, 3.414)	0.214
Tertile 3	1.000	(0.493, 2.029)	1.000	1.199	(0.552, 2.626)	0.646
	Factor 4: Fruits–nuts					
Continuous	0.982	(0.732, 1.313)	0.899	0.957	(0.696, 1.315)	0.787
Tertile 1	Ref			Ref		
Tertile 2	0.823	(0.404, 1.669)	0.589	0.884	(0.41, 1.901)	0.751
Tertile 3	1.477	(0.729, 3.019)	0.281	1.472	(0.685, 3.192)	0.323
	Factor 5: Staple food					
Continuous	1.246	(0.932, 1.686)	0.144	1.160	(0.834, 1.63)	0.382
Tertile 1	Ref			Ref		
Tertile 2	1.140	(0.56, 2.328)	0.717	0.955	(0.428, 2.127)	0.911
Tertile 3	2.348	(1.15, 4.883)	**0.020**	2.246	(1.013, 5.085)	**0.048**

^1^ unadjusted model. ^2^ adjusted model, adjusted for age, gender, BMI, daily screen time, educational level of the parents, family income, and parent–child relationship. *p*-Values < 0.05 are bolded.

**Table 4:** Eating behavior characteristics of ADHD and control children [Median (P25, P75)].

CEBQ	ADHD (n = 102)	Controls (n = 102)	Z	*p*-Values
Food avoidant dimension				
Satiety responsiveness	8 (6, 11)	8 (6, 11)	−0.207	0.836
Slowness in eating	6 (4, 9)	6 (4, 9)	−0.039	0.969
Food fussiness	10 (7, 12)	10 (8, 12)	−0.355	0.723
Emotional undereating	7 (5, 9)	7 (5, 8)	−0.926	0.354
Food approach dimension				
Food responsiveness	6 (3, 10)	4 (2, 9)	−1.661	0.097
Enjoyment of food	8 (5, 12)	8 (5, 11)	−0.673	0.501
Desire to drink	5 (3, 8)	4 (2, 7)	−2.248	**0.025**
Emotional overeating	2 (0, 4)	2 (0, 4)	−0.297	0.766

*p*-Values < 0.05 are bolded.

**Table 5:** Associations between eating behavior characteristics and the risk of ADHD.

Eating Behaviors	Model 1 ^1^			Model 2 ^2^		
	OR	95% CI	*p*-Values	OR	95% CI	*p*-Values
	Satiety responsiveness					
Continuous	0.986	(0.906, 1.073)	0.747	1.013	(0.922, 1.114)	0.789
Lower median	Ref			Ref		
Upper median	0.958	(0.538, 1.703)	0.883	1.085	(0.578, 2.045)	0.800
	Slowness in eating					
Continuous	0.995	(0.908, 1.089)	0.908	1.028	(0.926, 1.142)	0.604
Lower median	Ref			Ref		
Upper median	0.74	(0.414, 1.315)	0.305	0.861	(0.451, 1.643)	0.648
	Food fussiness					
Continuous	1.04	(0.951, 1.138)	0.392	1.052	(0.957, 1.159)	0.300
Lower median	Ref			Ref		
Upper median	1.24	(0.698, 2.211)	0.464	1.34	(0.721, 2.508)	0.357
	Emotional undereating					
Continuous	1.06	(0.971, 1.159)	0.199	1.064	(0.966, 1.175)	0.209
Lower median	Ref			Ref		
Upper median	1.138	(0.64, 2.026)	0.66	1.034	(0.544, 1.962)	0.917
	Food responsiveness					
Continuous	1.03	(0.968, 1.096)	0.356	1.009	(0.941, 1.082)	0.812
Lower median	Ref			Ref		
Upper median	1.609	(0.904, 2.882)	0.108	1.381	(0.721, 2.659)	0.330
	Enjoyment of food					
Continuous	1.031	(0.958, 1.11)	0.422	1.009	(0.929, 1.095)	0.835
Lower median	Ref			Ref		
Upper median	1.044	(0.587, 1.857)	0.883	0.811	(0.422, 1.544)	0.526
	Desire to drink					
Continuous	1.094	(1.003, 1.196)	**0.044**	1.083	(0.987, 1.190)	0.095
Lower median	Ref			Ref		
Upper median	2.125	(1.220, 3.738)	**0.008**	2.075	(1.137, 3.830)	**0.018**
	Emotional overeating					
Continuous	1.011	(0.912, 1.121)	0.835	0.971	(0.867, 1.087)	0.609
Lower median	Ref			Ref		
Upper median	0.958	(0.538, 1.703)	0.883	0.826	(0.442, 1.537)	0.548

^1^ unadjusted model. ^2^ adjusted model, adjusted for age, gender, BMI, daily screen time, educational level of the parents, family income, and parent–child relationship. *p*-Values < 0.05 are bolded.

## Data Availability

The datasets used and/or analyzed during the current study are available from the corresponding author upon reasonable request.

## Supplementary Materials

The following supporting information can be downloaded at: https://www.mdpi.com/article/10.3390/nu15051254/s1, Table S1: Dietary nutrient intake of ADHD and control group children [Median (P_25_, P_75_)].
