# Supplementary material for: Processed Food–Sweets Patterns and Related Behaviors with Attention Deficit Hyperactivity Disorder among Children: A Case–Control Study
Source: Nutrients. 2023 Mar 2;15(5):1254. doi: 10.3390/nu15051254 (PMC10005288; doi:10.3390/nu15051254)
Supplement: Supplementary file 1 [file nutrients-15-01254-s001.zip › nutrients-2217417-supplementary.pdf]

Table S1. Dietary nutrient intake of ADHD and control group children [Median (P_{25}, P_{75})]

**Dietary nutrient intake**	**RNI**	**ADHD (n=102)**	**Controls (n=102)**	***Z***	***Pp*-Values**
Energy (Kcal/d)	1400-2350	1771.1 (1404.9, 2005.8)	1510.2 (1315.2, 1757.0)	-2.755	**0.006**
Protein (g/d)	40-60	69.5 (53.1, 86.7)	66.3 (55.0, 71.6)	-1.735	0.083
Fat (g/d)	42.0-73.1	68.7 (57.6, 87.4)	63.4 (53.8, 76.8)	-2.216	**0.027**
Carbohydrate (g/d)	222-263	196.8 (158.3, 244.2)	166.7 (145.3, 211.3)	-2.586	**0.010**
Calcium (mg/d)	1000-1200	499.4 (357.1, 649.3)	551.7 (384.8, 728.8)	-1.386	0.166
Iron (mg/d)	13-18	15.4 (12.5, 19.7)	14.4 (12.4, 17.5)	-1.197	0.231
Zinc (mg/d)	7-12	10.9 (8.7, 13.9)	10.4 (8.3, 12.6)	-0.902	0.367
Selenium (μg/d)	25-55	38.9 (29, 51.6)	35.8 (31.9, 43.2)	-1.412	0.158
Phosphorus (mg/d)	470-710	1054.9 (798.5, 1217.7)	942.3 (758.1, 1096.7)	-1.685	0.092
Iodine (μg/d)	90-120	29.0 (19.1, 40.5)	22.0 (17.0, 32.0)	-2.656	**0.008**
Nicotinic acid (mg NE/d)	10-16	17.4 (13.0, 22.3)	15.6 (12.1, 18.9)	-2.193	**0.028**
Folic acid (μg DFE/d)	250-400	135.6 (89.9, 187.5)	130.6 (96.5, 190.9)	-0.407	0.684

RNI: recommended nutrient intake. *p*-Values <0.05 are bolded.
